# Consensus and experience trump leadership, suppressing individual personality during social foraging

**DOI:** 10.1126/sciadv.1600892

**Published:** 2016-09-14

**Authors:** Nicholas D. McDonald, Sean A. Rands, Francesca Hill, Charlotte Elder, Christos C. Ioannou

**Affiliations:** School of Biological Sciences, University of Bristol, Bristol BS8 1TQ, U.K.

**Keywords:** Consensus, coordination, self-organization, predation risk, refuge use, conformity, leadership, personality, boldness

## Abstract

Whether individual behavior in social settings correlates with behavior when individuals are alone is a fundamental question in collective behavior. However, evidence for whether behavior correlates across asocial and social settings is mixed, and no study has linked observed trends with underlying mechanisms. Consistent differences between individuals in boldness, which describes willingness to accept reward over risk, are likely to be under strong selection pressure. By testing three-spined sticklebacks (*Gasterosteus aculeatus*) in a risky foraging task alone and repeatedly in shoals, we demonstrate that the expression of boldness in groups is context-specific. Whereas personality is repeatable in a low-risk behavior (leaving a refuge), the collectively made consensus decision to then cross the arena outweighs leadership by bolder individuals, explaining the suppression of personality in this context. However, despite this social coordination, bolder individuals were still more likely to feed. Habituation and satiation over repeated trials degrade the effect of personality on leaving the refuge and also whether crossing the arena is a collective decision. The suppression of personality in groups suggests that individual risk-taking tendency may rarely represent actual risk in social settings, with implications for the evolution and ecology of personality variation.

## Teaser

A variety of mechanisms express and suppress individual behavioral tendencies during group decision-making in fish.

## INTRODUCTION

Living in groups is a widespread adaptation to avoid predators and exploit resources, and this has evolved independently in a diverse range of taxa (*1*, *2*). Despite the benefits of grouping, there is widespread variation in age, sex, and reproductive state and in less overt traits, such as tendencies to take risks (that is, boldness), between individuals, generating different preferences in the timing, location, and type of behaviors performed. This can incur “consensus costs” for individuals that have to suppress their preferences to stay with the group (*3*). The importance of within-group variation is evident in the wide range of processes that it can affect, including leadership (*4*), assortment by phenotype (*5*), reproduction (*6*), and dispersal (*7*).

Personality in animals is one such source of interindividual variation, broadly defined as consistent behavioral differences between individuals across time and contexts (*8*). The boldness-shyness continuum is one of the best understood personality traits and describes the willingness of individuals to accept greater risk (*9*) in return for a greater reward (*10*). It is becoming increasingly apparent that consistent individual variation in risk-taking has ecological and evolutionary effects (*11*). For example, individual variation in activity levels directly affects encounter rates between predators and prey (*12*) and, hence, has implications for predator-prey dynamics. Models of the adaptive significance of personality variation demonstrate that personality can reflect an individual-specific optimal balance between risk and reward (*13*). Although the relative differences between individuals in this trade-off would be expected to carry over from asocial to social settings, a number of mechanisms may affect this. Bolder individuals often lead group movements (*10*), and positive social feedback between leader and follower roles may stabilize and magnify these individual differences (*14*), resulting in a strong positive correlation between asocial and social behavior. In contrast, the need for group cohesion may drive consensus and coordination that reduces variation between individuals and suppresses the expression of individual personality in groups (*15*). However, the actual mechanism(s) driving conformity at the level of the collective are rarely demonstrated (*16*).

Studies that have explored the scaling of behavior from asocial to social settings have shown mixed results (*15*). Some have found positive correlations in individual behavior when tested alone and in groups (*17*, *18*), although this is not always the case (*19*, *20*). Whether individual behavioral traits are expressed in groups can vary depending on environmental conditions (*21*) and even for different behaviors under the same conditions (*22*). For example, in a recent study of fish swimming behavior (*16*), the consistent and repeatable variance in swimming speed of individuals was not expressed when the fish were tested in groups, but the median speed and turning speed remained correlated. Individual swimming traits were also less evident as group size increased from two to eight individuals, demonstrating conformity (*16*). However, even in moderately sized groups of a highly social fish (the golden shiner, *Notemigonus crysoleucas*), more motivated trained individual fish were still able to express this motivation when in groups with eight uninformed individuals (*23*), showing that conformity did not outweigh individual variation between trained individuals. Despite this work, no previous study has tested for the correlation in behavior when individuals are alone and in groups and then determined the mechanism(s) that explain the observed trends.

Here, we used three-spined sticklebacks (*Gasterosteus aculeatus*) and a simple foraging task to explore key factors linking behavior when tested alone to when the fish were randomly assembled into eight shoals of 10 fish. All trials took place in a 1.4-m × 0.7-m arena (fig. S1) where the fish started from a refuge at one end and could cross an open area to reach a conspicuous food stimulus that was visible from the refuge. The two behaviors measured varied in their degree of risk: the latency to first leave the refuge allows individuals to remain close to the refuge, whereas the latency to cross the open arena (being within three body lengths of the food stimulus, timed from when the refuge was left for the first time) is a riskier context due to greater exposure. We focus on refuge use because it is commonly used to assess boldness [including being shown to be repeatable in sticklebacks (*14*, *24*)], has been used to investigate collective dynamics (*25*), and has ecologically important impacts on predator-prey interactions (*26*) by affecting functional responses (*27*) and prey growth rates and fecundity (*28*). Fish were tested alone on day 1, in groups on days 2 and 3, and again alone on day 4. In group trials, the shoals were tested repeatedly for up to 10 trials in a day to explore the cumulative effect of training, habituation, and satiation as groups fed within a patch over time. This allowed us to test how group behavior, such as the degree of consensus and cohesion, changed because of these processes, as well as their effect on the correlation between asocial and social settings. This was repeated on the following day to examine whether these patterns changed after the fish were experienced with the arena but had lost any satiation.

## RESULTS

### Behaviors are repeatable in asocial settings

Behavior of individuals when tested alone was correlated across behaviors (leaving the refuge or crossing the arena) within trials and across days within behaviors (that is, between the first and second trials of testing fish alone) (fig. S2, A to D). As expected, the sum of these two latencies (the total time taken to reach the food) was also repeatable across trials (fig. S2E).

### Effect of social setting depends on behavior, and habituation depends on social setting

**Fig. 1:**
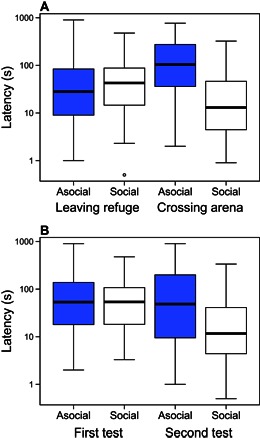
Factors affecting the latencies to leave the refuge and cross the arena for fish tested alone (blue) and the first trial on each group testing day (white). (**A**) The two latencies as a function of whether fish were tested alone (asocial) or in groups (social). There was a significant interaction between the behavior and social setting [negative binomial generalized linear mixed model (GLMM): deviance_11,12_ = 46.32, *P* = 1.004 × 10^−11^]. (**B**) There was also a significant interaction between latencies for fish tested alone or in groups and whether the trial was on the first or second day of testing within each of these treatments (deviance_11,12_ = 35.02, *P* = 3.26 × 10^−9^). The thick black lines represent the medians, the boxes encompass the interquartile ranges, the whiskers extend to the most extreme data points within 1.5× the interquartile range outside the box, and the circles show data points beyond the whiskers. Because only the first trial on each group testing day is included, the fish should be relatively hungry in both asocial and social trials as they were fed the previous day. The latencies are plotted on a log_10_ scale.

In the first trial of each fish on each day, before any of the fish could have consumed food and thus had similar levels of hunger, being in a group had little effect on the latency to first leave the refuge, whereas the fish were on average faster to cross the arena when tested in groups compared to when tested alone (Fig. 1A). This interaction also shows that, for the fish tested alone, they took longer to cross the arena than to leave the refuge, but this pattern was reversed when the fish were tested in groups. There was also a highly significant interaction between whether the fish were tested alone or in groups and whether it was the first or second day of testing under each of these treatments (Fig. 1B). Despite the trials with the fish tested alone being separated by 3 days, their average latencies were similar in both trials. In contrast, they were faster in the second group trial compared to the first (separated by only 1 day), suggesting that the fish were habituated only in a social setting.

### Correlations between testing alone and in groups depend on context

**Fig. 2:**
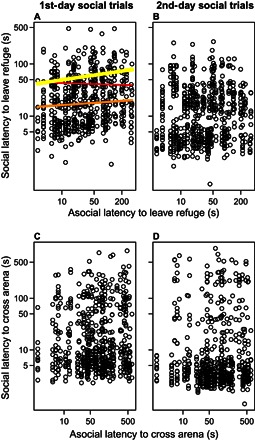
Relationship between latencies of fish tested alone (asocial) and in groups (social) for all group trials. (**A**) The relationship in the latency to first leave the refuge between asocial and social testing on the first day of group tests. The trend lines, fitted from the GLMMs, demonstrate the interaction between the asocial latency to leave the refuge and trial order (negative binomial GLMM: latency when tested alone × trial order: deviance_11,12_ = 6.44, *P* = 0.011), with the line thickness and color representing the trial number (with the first being the thick yellow line, the fifth being the thinner orange line, and the tenth being the thin red line). Only these trend lines are shown for clarity (note that the trendlines for the other orders are intermediate between these as trial order is a continuous variable). (**B**) Same relationship on the second day of group trials. (**C** and **D**) Relationship in the latency to cross the arena between asocial and social settings on the first (C) and second (D) days of group trials. There are no significant relationships between asocial and social latencies in (B) to (D) (table S1). All latencies are plotted on a log_10_ scale.

To avoid the possible confounding effect of experience gained between days 1 and 4, we used the two latencies on the first day as measures of the fish’s boldness to predict the corresponding latency in group trials. Initially, there was a positive association between leaving the refuge when tested alone and leaving the refuge in group trials. This effect diminished as trials progressed on the first day of group trials and even had a negative slope at the end of the trials (Fig. 2A). On the second day of group testing, there was no significant association or interaction with trial order (Fig. 2). These trends indicate the need to consider other variables, such as satiation (*29*), in examining the relationship in a behavior between asocial and social settings. There was no association in the latency to cross the arena between asocial and social settings on either day of group trials (Fig. 2, C and D). Reflecting these results for the latency to cross the arena, the total time taken to reach the food (the sum of the two former latencies) in group trials was also not associated with the total time taken when the fish were tested alone (table S1).

### Other effects on latencies are similar between behaviors

**Fig. 3:**
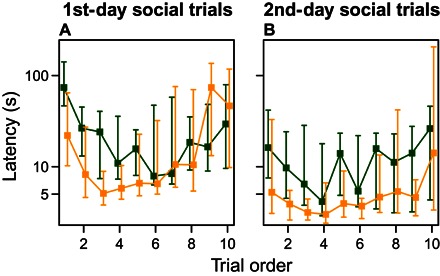
Change in the latency to leave the refuge (dark green) and cross the arena (yellow) as group trials progressed. Effect of trial order within the (**A**) first day of group trials and (**B**) second day of group trials. All trends show a significant polynomial effect (negative binomial GLMM: polynomial latency to leave the refuge: first day: deviance_10,11_ = 194.12, *P* < 2.2 × 10^−16^; second day: deviance_9,10_ = 405.9, *P* < 2.2 × 10^−16^; polynomial latency to cross arena: first day: deviance_9,10_ = 105, *P* < 2.2 × 10^−16^; second day: deviance_9,10_ = 102.26, *P* < 2.2 × 10^−16^). Medians and interquartile ranges are shown. The latencies are plotted on a log_10_ scale.

Despite these differences between the latencies to leave the refuge and to cross the arena, explanatory variables other than the corresponding latency when tested alone had similar effects on the two latencies in group trials (table S1). On both days, the fish became faster over repeated trials until approximately halfway through the 10 trials before slowing down again (Fig. 3). This is expected from an initial training/habituation phase (*30*) followed by satiation, increasing the time taken (*31*). The fish that fed in the previous trial were faster than the other fish in both leaving the refuge and crossing the arena, which is also suggestive of training (fig. S3 and table S1).

### Mechanisms: Consensus decision-making

**Fig. 4:**
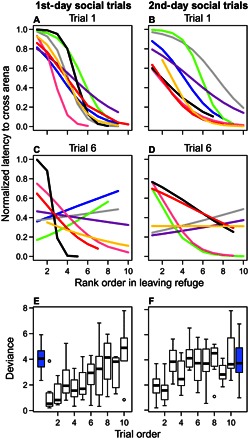
Collective decision to cross the arena and its decline over repeated trials. (**A** and **B**) Rank order of the fish in their latency to first leave the refuge is related to their latency to then cross the arena for the first trial in the first (A) and second (B) days of group trials. Logistic regressions are fitted for each trial separately. (**C** and **D**) Same relationship for the sixth trial on the first (C) and second (D) day of group trials. Line colors represent different groups and correspond across panels (including fig. S5 and S6). (**E** and **F**) Change in the goodness of fit of these relationships across each day on the first (E) and second (F) days of group trials (boxes, whiskers, etc. as in Fig. 1). The deviance of the model fits increases significantly as the trials progress (Poisson GLMM: first day: deviance_3,4_ = 29.16, *P* = 6.65 × 10^−8^; second day: deviance_3,4_ = 8.84, *P* = 0.0029). The deviances of the model fits for the same analysis for each group applied to the latencies when fish were tested alone on the first day before the group testing [blue box in (E)] and when tested alone on the last day after group testing [blue box in (F)] are also shown.

We explored the link between the latencies in leaving the refuge and crossing the arena to quantify the extent to which the decision to cross the arena was a collective decision. Using logistic regression, the relationship in each trial between each fish’s rank order of first leaving the refuge and their latency to cross the arena was determined (*32*). Logistic regression provides a simple fit that can capture the nonlinear relationship often found between these variables (for example, as typical of quorum group decision-making). We used the deviance as a measure of fit, which is a measure of the variance of the observed data around the fitted curve. At the start of the group testing, the fish showed a strong collective response (Fig. 4, A and B), with the latency to cross the arena decreasing rapidly as the number of fish that had already left the refuge for the first time increased. However, as the trials progressed, this relationship became less and less strong (Fig. 4, C and D, and figs. S4 and S5), supported by an increasing deviance of the model fits (Fig. 4, E and F) and greater variation between groups (figs. S4 and S5). This demonstrates that the decision to cross the arena was less collective as the trials progressed and as the fish became more habituated and satiated. Thus, the fish became less dependent on social interactions when foraging as the trials progressed (*33*). For the last trials on each day, the model fits from the group trials were similar to a “null,” nonsocial expectation based on the same analysis applied to the latencies when the fish were tested alone before and after group trials (Fig. 4, E and F).

### Mechanisms: Cohesion

The pattern seen in collective decision-making and the difference in the two latencies in whether there was a relationship between asocial and social settings can be explained by the fish crossing the arena as a cohesive shoal, with greater conformity between the fish than when leaving the refuge. If this is the case, the fish should be more clustered in time, having a lower variability between fish (*16*) when they reach the food compared to when they leave the refuge. For the first trials on each of the group trials, the variability of times to reach the food within each trial was lower than the variability in leaving the refuge (fig. S6), that is, the fish were closer to one another in time. There was also an effect of trial order within a day; as the trials progressed, the difference between latencies was reduced.

This trend was confirmed at the individual level by the minimum time delay from each fish’s latency to another fish’s latency in the trial, calculated separately for times to leave the refuge and to reach the food. For the first fish in each trial, the time delay to another fish was shorter when reaching the food compared to leaving the refuge (fig. S7, A and B). As more fish performed each behavior, both delays decreased until they converged, with the last fish having longer delays in reaching the food. Trial order also had a significant effect on the first day, with the delays in the first trials between fish in leaving the refuge being longer than the delays in reaching the food. Again, the difference decreased as the trials progressed, and the differences between the two latencies were not observed on the second day of group trials (fig. S7, C and D). These trends demonstrate that the fish were more cohesive when reaching the food than when leaving the refuge if potential risk would have been perceived to be high, namely, when they were the first fish to reach the food within a trial and also when the fish had less experience with the arena. Compared to a null, nonsocial expectation where these analyses of cohesion were repeated on the same group memberships but using latencies when the fish were tested alone, there was strong evidence that the fish were more cohesive than expected by chance (figs. S6 and S7).

### Mechanisms: Leadership driven by boldness

To further elucidate the role of social interactions in the expression of personality between asocial to social settings, the *x* and *y* coordinates of each fish were recorded from the still image of the video at the point the first fish crossed the arena and reached the food in each group trial. At this point in the trials, there was a significant association between a fish’s boldness and its distance from the food stimulus (fig. S8). Bolder fish were closer to the food stimulus, suggesting that they lead the group movement across the arena. Previous studies have shown that frontal positions in groups are associated with leadership (*34*). However, the lack of correlation between asocial and social settings for the latency to cross the arena (from first leaving the refuge) and the total time taken to reach the arena (from the trial starting) suggests that this effect was outweighed by the coordination between fish in crossing the arena.

### Rank order within trials is affected by boldness

In addition to analyzing the absolute latencies in leaving the refuge and crossing the arena, we also examined the rank order of individuals’ latencies in each group trial. The order within each group of leaving the refuge when the fish were tested alone was positively related to their order of leaving the refuge in the group trials (fig. S9). This effect did not change over the group trials on the first day, unlike the latency times to leave the refuge where the positive relationship was lost as trials progressed (Fig. 2A), and was still evident on the second day of group trials.

In contrast, the relationship between the rank order of crossing the arena in asocial and social trials did change as the trials progressed on the first day of group trials. Initially, there was actually a negative association of ranks between asocial and social trials (fig. S9C). This effect can be explained by the collective and coordinated response in crossing the arena and leadership by bolder individuals: bolder individuals left the refuge first (fig. S9A) but were delayed in crossing the arena until a consensus was reached where enough of the group decided to follow. This resulted in relatively longer times to cross the arena for bolder fish compared to shyer individuals, which left the refuge later but crossed the arena with less delay as they were led by the bolder fish. The negative relationship became weaker and then became positive as the trials progressed on the first day of group trials, and there were no significant trends on the second day of group trials (fig. S9D), possibly because the collective response was weaker as the fish became habituated and sated.

### Bolder fish are more likely to feed

**Fig. 5:**
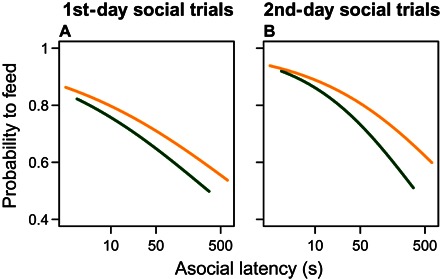
Probability of feeding in group trials as a function of individuals’ latency to leave the refuge (dark green) and cross the arena (yellow) when tested alone (that is, their boldness). Fitted lines are calculated from the GLMM fixed-effect coefficients for the first (**A**) and second (**B**) day of group trials. Bolder individuals were significantly more likely to feed in all cases (binomial GLMM: first day, latency to leave refuge: deviance_7,8_ = 6.53, *P* = 0.011, latency to cross arena: deviance_7,8_ = 5.91, *P* = 0.015; second day, latency to leave refuge: deviance_7,8_ = 11.37, *P* = 0.00075, latency to cross arena: deviance_7,8_ = 7.73, *P* = 0.0054). The models are carried out separately for each day and with each latency as the explanatory variable as boldness (that is, four models in total; table S1). The latencies are plotted on a log_10_ scale.

Because of the effect of boldness on distances to the food stimulus and their rank order of reaching the food, we explored whether bolder fish were more likely to feed in each trial (*10*, *24*) and whether this effect changed as the group trials progressed. On both days of the group trials, bolder individuals were more likely to feed in each trial (Fig. 5 and table S1), and this effect was not significantly affected by the order of the trial in each day. Thus, although the effects of consensus decision-making and experience suppressed a correlation in crossing the arena from the asocial to social setting, bolder fish were still able to feed more than shyer fish while benefiting from group cohesion.

## DISCUSSION

Our study reveals that the scaling of individual behavior from asocial to social settings is multifaceted. In leaving the refuge, individuals were repeatable between testing alone and testing in groups, at least at the start of each day when hunger was more even across the group and the arena was less familiar. However, there was no significant association between asocial and social settings in the latency to then cross the arena. The strong collective and cohesive response in crossing the arena could explain this trend, which also explained why the within-trial rank order of latencies to cross the arena was negatively correlated between asocial and social settings. Despite this loss of repeatability in the latency to cross the arena, personality was still evident as bolder individuals appeared to lead group movements and were more likely to consume food once the food was reached. Thus, consensus decision-making and leadership by bolder individuals are not mutually exclusive, although it appears that the collective response has the stronger effect and results in a lack of repeatability in crossing the arena. Our results link a number of previous studies on collective behavior and personality in a social setting, allowing us to determine the mechanisms that are likely to underpin whether personality is expressed in groups.

The type of situation our fish were tested in, where they could either remain in a safe patch without food or risk exposure to predators to feed, is a major paradigm in both experiments and theory (*14*, *35*). Moreover, it is widespread in natural environments and has important impacts on ecological processes such as predator-prey interactions because it can determine the availability of prey to predators (*26*, *27*). In the terms of our experiment, predation risk would be much greater when crossing the arena compared to when leaving the refuge because leaving the refuge only required the fish to be partially outside of cover. This is consistent with the finding that being in a group reduced the latency to cross the arena, as grouping is frequently associated with a decreased perceived risk of predation (*18*, *36*). Thus, we may expect that the latency to cross the arena is more tightly regulated by the boldness of an individual than leaving the refuge. In this case, bolder individuals would lead groups from the front (*10*, *14*) and cross the arena sooner. Although the bolder fish were closer to the food stimulus when the arena was first crossed in each trial, suggestive of leadership, the effects of consensus and coordination were sufficiently strong to suppress a correlation in the time taken to cross the arena between asocial and social settings. This lack of predictability suggests that behavior when tested alone, despite its repeatability, is unlikely to be predictive of the risk actually experienced by an individual when in a social setting. Determining the interactions between risk-taking personality and evolutionary or ecological processes in social species may thus be difficult, and there may be fundamentally different mechanisms for selection on personality traits in social animals (*35*, *37*) compared to mechanisms that apply to both social and nonsocial species [for example, see study by Wolf *et al.* (*13*)]. Alternatively, in other situations or species, motivation may vary enough between individuals within groups that the groups fragment (*23*, *38*), which would be expected to reinstate correlations of individual behavior between asocial and social settings.

The two latencies initially showed different responses to sociality; however, the multiple trials as a group had little effect on these behaviors when the fish were retested alone. Both latencies were, on average, similar between the 2 days of single-fish trials, suggesting that the training that occurred in group trials (evident in the faster latencies in the first trial of the second day of group trials compared to the first day) did not carry over to the final day of testing. The repeatabilities of the two latencies between pre- and postgroup trials were also similar, as was the correlation between the two latencies within each day of testing fish alone. These trends are particularly surprising given that testing of fish alone was separated by 3 days, whereas all other comparisons (that is, between asocial and social settings) took place at a shorter time scale. It appears that the difference between the two latencies in their association from asocial to social settings is a plastic response to the social environment, with group testing having no lasting effects on the averages of, the repeatability of, or the relationship between the two behaviors. Thus, there was no evidence that the repeated social testing resulted in a convergence (*6*) or differentiation [social niche specialization (*39*)] in consistent variation between individuals when tested alone. In previous work in an asocial context, bolder individuals have been shown to learn more quickly (*40*), and experience can increase interindividual variation, for example, due to more exploratory individuals learning to habituate more quickly to novel environments (*41*); further work on the interaction between personality, experience, and sociality is clearly warranted.

Although conformity is well documented in animal groups (*15*), the mechanisms behind the conformity are often undetermined [for example, see studies by Herbert-Read *et al*. (*16*) and Stienessen and Parrish (*42*)]. Our study shows experimentally that conformity in crossing the arena (reducing variation between individuals) is driven by quorum-like consensus decision-making (*43*), providing a self-organized mechanism for the loss of individual expression in groups. However, this collective decision-making appeared to reduce as the trials progressed, as did the cohesion between individuals in their times to cross the arena. Therefore, it could be expected that once the fish were less collective in crossing the arena, their individual preferences as measured when tested alone would correlate with their latency to cross the arena in group trials. There was some evidence of this in the rank order of latencies to cross the arena in the latter group trials (red line in fig. S9C), although this was only observed on the first day of group trials and not in the raw latency values, only in their rank order. The strong overall trend in both latencies of a cumulative effect of training, habituation, and satiation can explain the lack of personality expression. We could not reliably estimate how well trained or sated each individual was in each trial, in part because the videos did not have enough resolution to allow us to determine which fish ate each bloodworm. However, previous work using pairs of sticklebacks where feeding was tightly controlled does demonstrate that both learning (*44*) and satiation (*29*) can interfere with the expression of personality in groups. The loss of an association in the latency to leave the refuge between asocial and social settings on the first day of group trials supports this explanation.

It is becoming increasingly clear that individual differences in personality can have important effects in groups, including determining group-level behavior (*25*, *45*), the benefits individuals gain from being in groups (*44*, *46*), and the distribution of influence between individuals on group decisions (*17*, *29*). Although we found some evidence of bolder individuals being more influential by being at the front of groups as they approach the stimulus, processes of group coordination and experience were strong enough to lose the correlation of individual behavior and, hence, suppress the expression of personality in groups. Identifying the mechanisms underlying this trend provides evidence for the relative strengths of within-group processes such as conformity and leadership, which may vary between species and contexts. Although commonly used in both personality and group decision-making studies (*9*, *24*, *44*), the species used here, the three-spined stickleback, is not the most social of animals; it is only facultatively social and uses less social information compared to closely related species (*47*). Thus, our results should apply more strongly to animals where group cohesion is even more important and, therefore, apply to a wide range to social species.

## MATERIALS AND METHODS

### Study design

The experiment was designed to quantify the behavior of individuals when tested alone (including the correlation in behavior before and after testing in a group) and to examine under what conditions this behavior was significantly related to the behavior of individuals when tested in groups. We used a relatively large group size (compared to similar studies) to allow for consensus decision-making and repeatedly tested fish in their groups within a day to explore cumulative effects of training, habituation, and satiation. This also allowed us to test whether correlations between asocial and social behaviors weakened or strengthened over repeated tests. Recording trials using video allowed us to quantify the timing of events accurately, to identify the fish using individually marked tags, and to record their coordinate positions relative to the food stimulus when the food was first reached in each trial. We also measured whether each fish ate at least one bloodworm in each group trial.

### Experimental subjects

Fish (mean ± SD; standard body length, 43 ± 3.2 mm) were caught from the River Cary, Somerset, UK (ST 469 303) and were held in 120-cm × 45-cm × 37.5-cm tanks for at least 3 months before testing. Water temperature was maintained at 15° to 16°C and a 10-hour:14-hour day/night photoperiod throughout. The fish were fed defrosted bloodworms and flake ad libitum daily, and only after testing on experimental trial days. Eighty fish were used in the study.

### Experimental protocol

Ten fish with standard body lengths within 5 mm of one another made up each of the eight groups, and two groups were tested concurrently. The fish were individually tagged [using a 5-mm numbered plastic disc over the middle spine (*48*)] and held in their groups in 16.5-cm × 12.7-cm × 12.7-cm breeding nets positioned within one of the stock tanks for 5 days before the start of testing.

The experimental arena (1.4 m × 0.7 m; water depth, 11 ± 1 cm) (fig. S1) was surrounded by a 1.8-m-high wooden frame and white sheets. The arena was filmed from above with a Panasonic SD800 camera at a resolution of 1920 × 1080. Two trapezoid pens at one end of the arena housed the fish before each trial, each of which was covered by a black plastic mesh (5 mm) to create a darkened refuge. One group was assigned to each of the two pens. Two pipettes were held vertically in white opaque tubes in the opposite corner of the pen to where the fish would start in that trial. Red polyvinyl chloride tape was wrapped around the end of one pipette to provide a 5-mm × 3-mm (length × diameter) stimulus visible past the end of the tube. This was positioned 15 cm from the side wall of the arena and was visible at any position within the pen; red on a white background is highly conspicuous to sticklebacks (*49*). Adjacent to this, the other pipette was positioned 17 cm from the side wall of the arena. The opaque tubes ensured that the bloodworms (two per fish in the trial) inside this pipette were not visible. Olfactory cues were minimized by placing an air bubble at the end of this pipette to form an air barrier between the water in the pipette and the arena.

On day 1, each of the 20 fish was tested in a randomized order. The fish were transferred to their allocated pen and allowed to habituate for 2 min before the door of the pen was raised. Two measures of boldness were recorded: the latency for the fish to first leave the refuge (defined as when their tag was first visible) and the latency for the fish to cross the arena from first leaving the refuge, measured as being within 13.4 cm of the pipette (this radius was marked on a monitor used to observe the trials and was approximately 3 × mean standard body length). Latencies were scored from video and were recorded blind in terms of the experimenters not knowing the behavior of individual fish in other trials. The bloodworms were pipetted into the arena when the radius was first crossed. If a fish had not left the refuge within 15 min, it was given a maximum latency to leave the refuge and total time to cross the arena of 15 min, and no latency was recorded for the latency to cross the arena from first leaving the refuge. This occurred four times when testing the fish alone, twice on the first day of single-fish trials, and twice on the second day. Trials were also terminated if the fish did not cross the arena within 15 min or if the bloodworms were not eaten within 20 min from starting the trial (6 fish on the first day of single-fish trials and 16 on the last day did not cross the arena after leaving the refuge). The fish were then returned to their breeding net. These single-fish trials were repeated again on the final day after 2 days of group testing.

On the day following the first day of testing fish alone, the groups of 10 fish were transferred to their pens to habituate 1 hour before testing. The procedure for the single-fish trials was repeated, with the latency to leave the refuge and to cross the arena being recorded for each fish in each trial. Whether each individual fed was also recorded, and the trial ended after all bloodworms were eaten. At the point in each trial that the first fish crossed the arena, the coordinates of each fish and the food stimulus were recorded manually from the still image from the video (using ImageJ). Again, the trials were terminated if no fish crossed the radius within 15 min or if not all of the bloodworms were eaten within 20 min. The fish were returned to their pen after each trial, whereupon the group in the other pen was tested. A maximum of 10 trials were carried out per day for each group; if a trial was terminated, then the group was not tested again that day (leading to 6 group trials from a possible total of 160 not being carried out). The groups were left overnight in their pens before repeating the group trials the following day. All trials took place between 10:30 a.m. and 4:30 p.m., from 4 October to 15 November 2013. In one group trial, the video was corrupted; thus, the data were lost. Frequencies of individuals per trial performing each behavior are given in table S2. All procedures regarding use of animals in research followed UK guidelines, which met international standards and were approved by the University of Bristol Ethical Review Group (UIN UB/11/042).

### Statistical analysis

Both latencies showed right-skewed distributions in single-fish and group trials; thus, they were log-transformed when used as explanatory variables and analyzed as response variables using negative binomial GLMMs. To determine the effects of being in a group and experience while minimizing the effect of differences in hunger, we compared latencies to leave the refuge and cross the arena in the first trial for each fish on each day. We used the latencies of fish when tested alone as an explanatory variable in the analysis of latencies in group trials to test whether latencies correlated between asocial and social settings. This was carried out separately for latencies to leave the refuge and cross the arena, and we included a polynomial effect of trial order per day (1 to 10) after visually inspecting the data (Fig. 3). This analysis was repeated using the rank order of latencies within each group both in group trials and when the same fish were tested alone [linear mixed models (LMMs) were used in this case; the residuals were approximately normally distributed]. From the coordinates of each fish when the food stimulus was first reached, the distance of each fish to the food stimulus was analyzed using negative binomial GLMMs, with measures of boldness as an explanatory variable (run separately for each day and type of asocial latency when tested alone). Using binomial GLMMs, we also analyzed the probability that each fish would eat at least one bloodworm in each group trial, again as a function of boldness.

Analyses of group cohesion and coordination were based primarily on group trial data. For the analysis of consensus decision-making, the latency to cross the arena for each fish was normalized within each trial to range from 0 (fastest fish) to 1 (slowest fish) and was expressed as a proportion of the time taken by the slowest fish as the response variable in the logistic regression. Only the fish that left the refuge before the arena was first crossed in that trial were included in this analysis. To analyze cohesion, for each fish, the minimum time delay to another fish first leaving the refuge and crossing the arena was analyzed using negative binomial GLMMs. A similar analysis was carried out at the group level by calculating the SD of latencies to leave the refuge and cross the arena in each group trial. To shed further light on these group dynamics, the same variables were calculated for each group but instead using the latencies of the fish in those groups when tested alone (both before and after the group trials). This gave a null, nonsocial benchmark of what would be expected from the same individuals in each group without the ability to interact socially.

Each model structure is presented in table S1 together with the coefficient for each term and their significance. All tests were two-tailed, the dispersion parameter was checked to be approximately 1 (between 0.5 and 2) for negative binomial, binomial, and Poisson tests, and all analyses were carried out in *R* version 3.0.2.

## Supplementary Material

http://advances.sciencemag.org/cgi/content/full/2/9/e1600892/DC1

## SUPPLEMENTARY MATERIALS

Supplementary material for this article is available at http://advances.sciencemag.org/cgi/content/full/2/9/e1600892/DC1 fig. S1. Overhead view of experimental apparatus. fig. S2. Repeatability within and between behaviors when fish were tested alone (that is, in an asocial setting). fig. S3. Effect of trial order and whether a fish fed in the previous trial on the latency to leave the refuge or cross the arena. fig. S4. Collective decisions to cross the arena and its change over repeated trials on the first day of group trials. fig. S5. Collective decisions to cross the arena and its change over repeated trials on the second day of group trials. fig. S6. SD of latencies to leave the refuge and the total time taken to reach the food within each trial as the group trials progressed each day. fig. S7. Minimum time delay from each fish to another fish in the trial to first leave the refuge and reach the food. fig. S8. Effect of boldness on the proximity of individuals to the food stimulus at the end of each group trial. fig. S9. Relationship between the rank order in each group of latencies of fish tested alone (asocial) and in groups (social) for all group trials. table S1. Summaries of the statistical models. table S2. Frequencies of fish per trial leaving the refuge, crossing the arena, and consuming food.
