# Supplementary material for: Consensus and experience trump leadership, suppressing individual personality during social foraging
Source: Sci Adv. 2016 Sep 14;2(9):e1600892. doi: 10.1126/sciadv.1600892 (PMC5023318; doi:10.1126/sciadv.1600892)
Supplement: http://advances.sciencemag.org/cgi/content/full/2/9/e1600892/DC1 [file supp_2_9_e1600892__index.html]

Science Advances | Science Advances

## Supplementary Materials

**This PDF file includes:**

- fig. S1. Overhead view of experimental apparatus.
- fig. S2. Repeatability within and between behaviors when fish were tested alone (that is, in an asocial setting).
- fig. S3. Effect of trial order and whether a fish fed in the previous trial on the latency to leave the refuge or cross the arena.
- fig. S4. Collective decisions to cross the arena and its change over repeated trials on the first day of group trials.
- fig. S5. Collective decisions to cross the arena and its change over repeated trials on the second day of group trials.
- fig. S6. SD of latencies to leave the refuge and the total time taken to reach the food within each trial as the group trials progressed each day.
- fig. S7. Minimum time delay from each fish to another fish in the trial to first leave the refuge and reach the food.
- fig. S8. Effect of boldness on the proximity of individuals to the food stimulus at the end of each group trial.
- fig. S9. Relationship between the rank order in each group of latencies of fish tested alone (asocial) and in groups (social) for all group trials.
- table S1. Summaries of the statistical models.
- table S2. Frequencies of fish per trial leaving the refuge, crossing the arena, and consuming food.

Download PDF

**Files in this Data Supplement:**

- Adobe PDF - 1600892\_SM.pdf
