# Supplementary material for: Consensus and experience trump leadership, suppressing individual personality during social foraging
Source: Sci Adv. 2016 Sep 14;2(9):e1600892. doi: 10.1126/sciadv.1600892 (PMC5023318; doi:10.1126/sciadv.1600892)
Supplement: http://advances.sciencemag.org/cgi/content/full/2/9/e1600892/DC1 [file 1600892_SM.pdf]

Science Advances

AAAS

advances.sciencemag.org/cgi/content/full/2/9/e1600892/DC1

Supplementary Materials for

Consensus and experience trump leadership, suppressing individual personality during social foraging

Nicholas D. McDonald, Sean A. Rands, Francesca Hill, Charlotte Elder, Christos C. Ioannou

Published 14 September 2016, *Sci. Adv.* **2**, e1600892 (2016)
DOI: 10.1126/sciadv.1600892

This PDF file includes:

- fig. S1. Overhead view of experimental apparatus.
- fig. S2. Repeatability within and between behaviors when fish were tested alone (that is, in an asocial setting).
- fig. S3. Effect of trial order and whether a fish fed in the previous trial on the latency to leave the refuge or cross the arena.
- fig. S4. Collective decisions to cross the arena and its change over repeated trials on the first day of group trials.
- fig. S5. Collective decisions to cross the arena and its change over repeated trials on the second day of group trials.
- fig. S6. SD of latencies to leave the refuge and the total time taken to reach the food within each trial as the group trials progressed each day.
- fig. S7. Minimum time delay from each fish to another fish in the trial to first leave the refuge and reach the food.
- fig. S8. Effect of boldness on the proximity of individuals to the food stimulus at the end of each group trial.
- fig. S9. Relationship between the rank order in each group of latencies of fish tested alone (asocial) and in groups (social) for all group trials.
- table S1. Summaries of the statistical models.
- table S2. Frequencies of fish per trial leaving the refuge, crossing the arena, and consuming food.

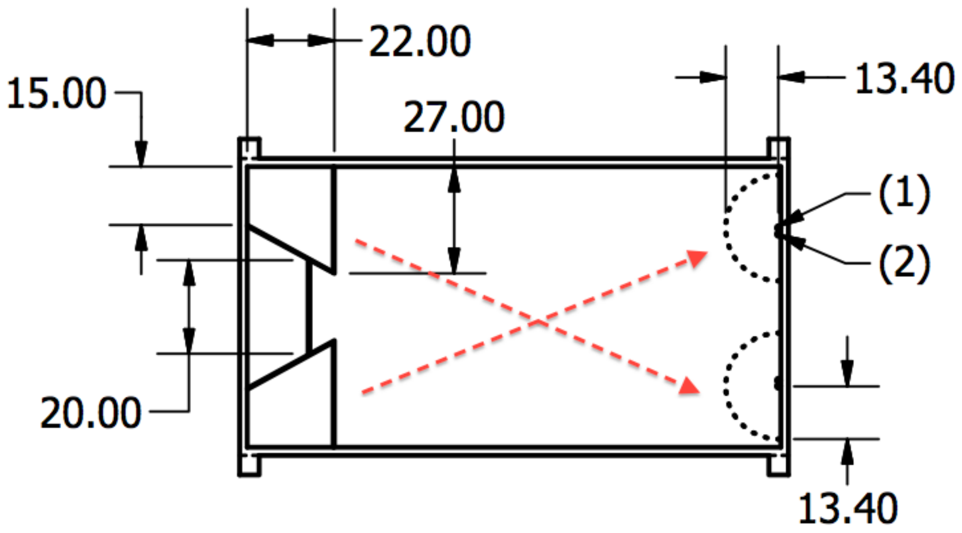

fig. S1. Overhead view of experimental apparatus. All measurements are in cm. Arena walls are indicated by the double solid lines, with single solid lines within the arena representing opaque walls; the two groups tested concurrently were not in visual contact with one another. (1) indicates the pipette with the standardized red stimulus, and (2) indicates the pipette through which the bloodworms are ejected. The dotted circular lines illustrate the radius fish had to cross before the bloodworms were ejected. The red dashed arrows illustrate the direction of the standardised stimulus from the starting pens.

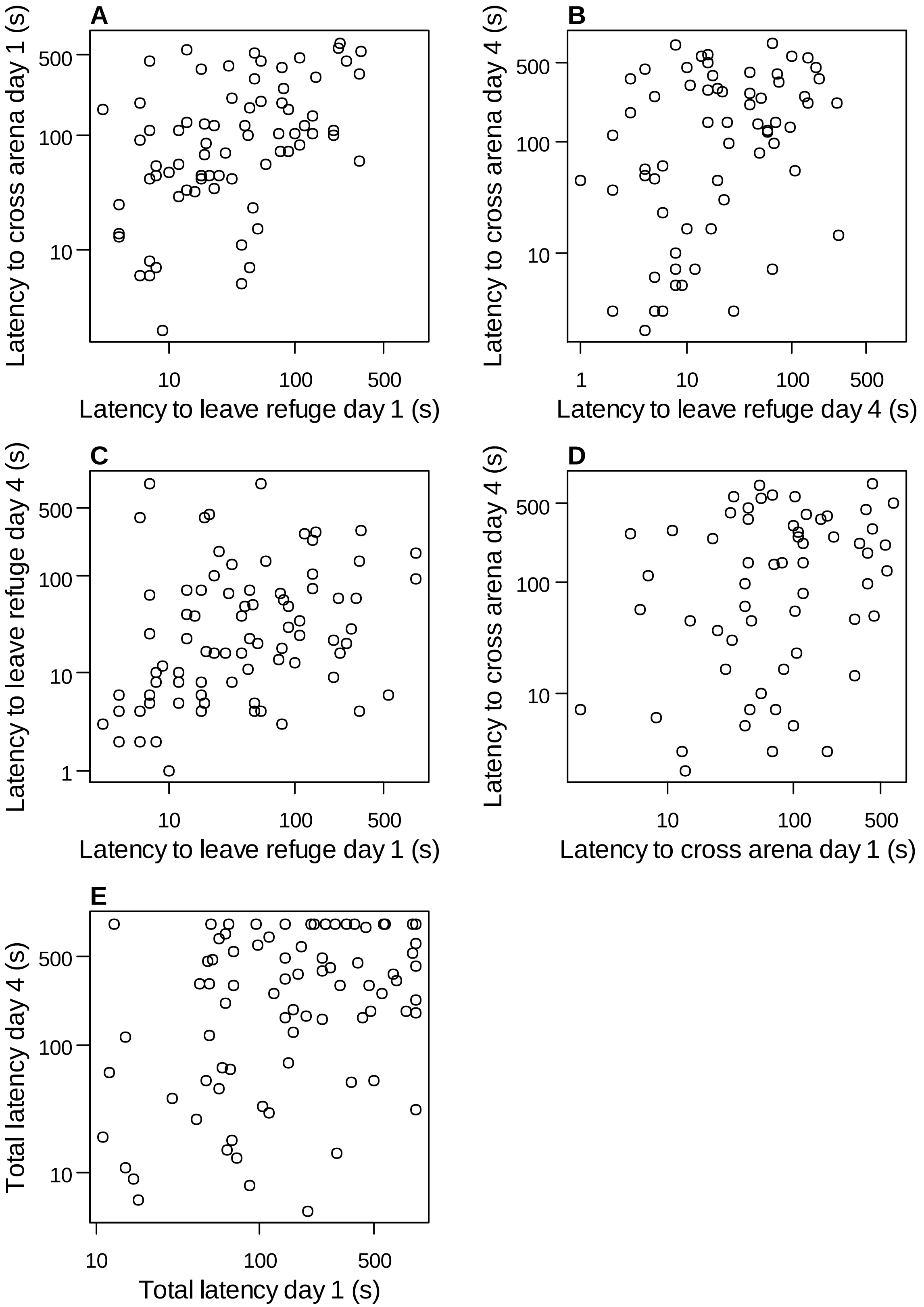
A
Latency to cross arena day 1 (s)
Latency to leave refuge day 1 (s)
B
Latency to cross arena day 4 (s)
Latency to leave refuge day 4 (s)
C
Latency to leave refuge day 4 (s)
Latency to leave refuge day 1 (s)
D
Latency to cross arena day 4 (s)
Latency to cross arena day 1 (s)
E
Total latency day 4 (s)
Total latency day 1 (s)

fig. S2. Repeatability within and between behaviors when fish were tested alone (that is, in an asocial setting). (**A**) The correlation between the latency to first leave the refuge and cross the arena on the first day of asocial trials (Spearman's rank correlation: $r_s = 0.49$, N = 74, P = 1.15 × 10^{-5}), and (**B**) the second ($r_s = 0.35$, N = 64, P = 0.0048). (**C**) The correlation between the first and second days of asocial trials in the latency to first leave the refuge ($r_s = 0.37$, N = 80, P = 0.00081), and (**D**) in the latency to first cross the arena ($r_s = 0.27$, N = 59, P = 0.036). (**E**) The correlation between the total latency to leave refuge and cross the arena between the first and second days of asocial trials ($r_s = 0.31$, N = 80, P = 0.0045). Both axes are on a log10 scale.

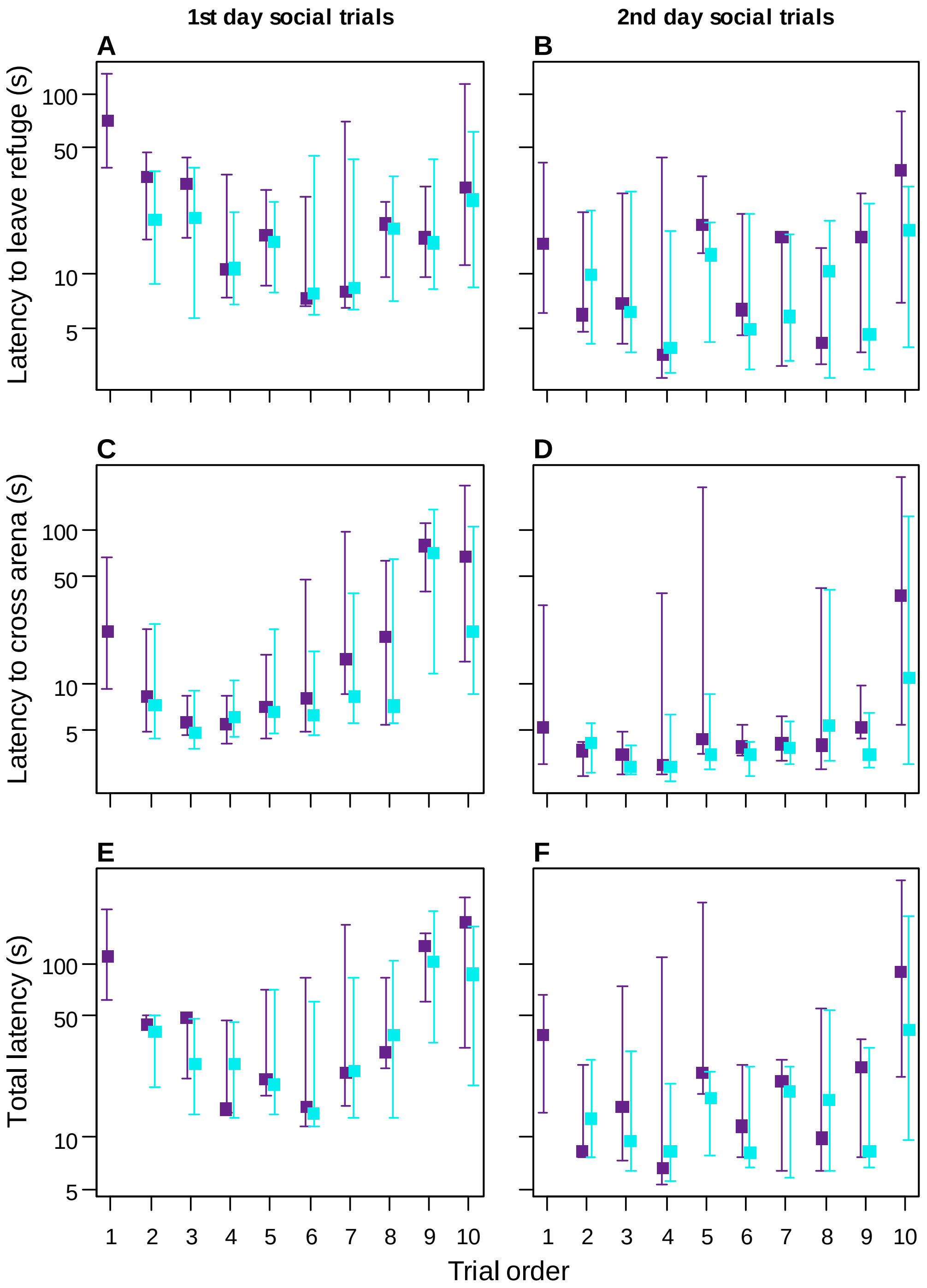

fig. S3. Effect of trial order and whether a fish fed in the previous trial on the latency to leave the refuge or cross the arena. Data from individuals that fed in the previous trial are represented in cyan, and those that did not in purple. Note for the first trial on each day, no fish could have fed in the previous trial. (**A**) The latency to first leave the refuge on the first day of group testing and (**B**) the second day. (**C,D**) The same comparisons for the latency to cross the arena, and (**E,F**) the total time taken (the total of the two latencies). In all cases there is a significant main effect of whether a fish fed in a previous trial (Table S1). Shown are medians and interquartile ranges. The latencies are plotted on a log10 scale.

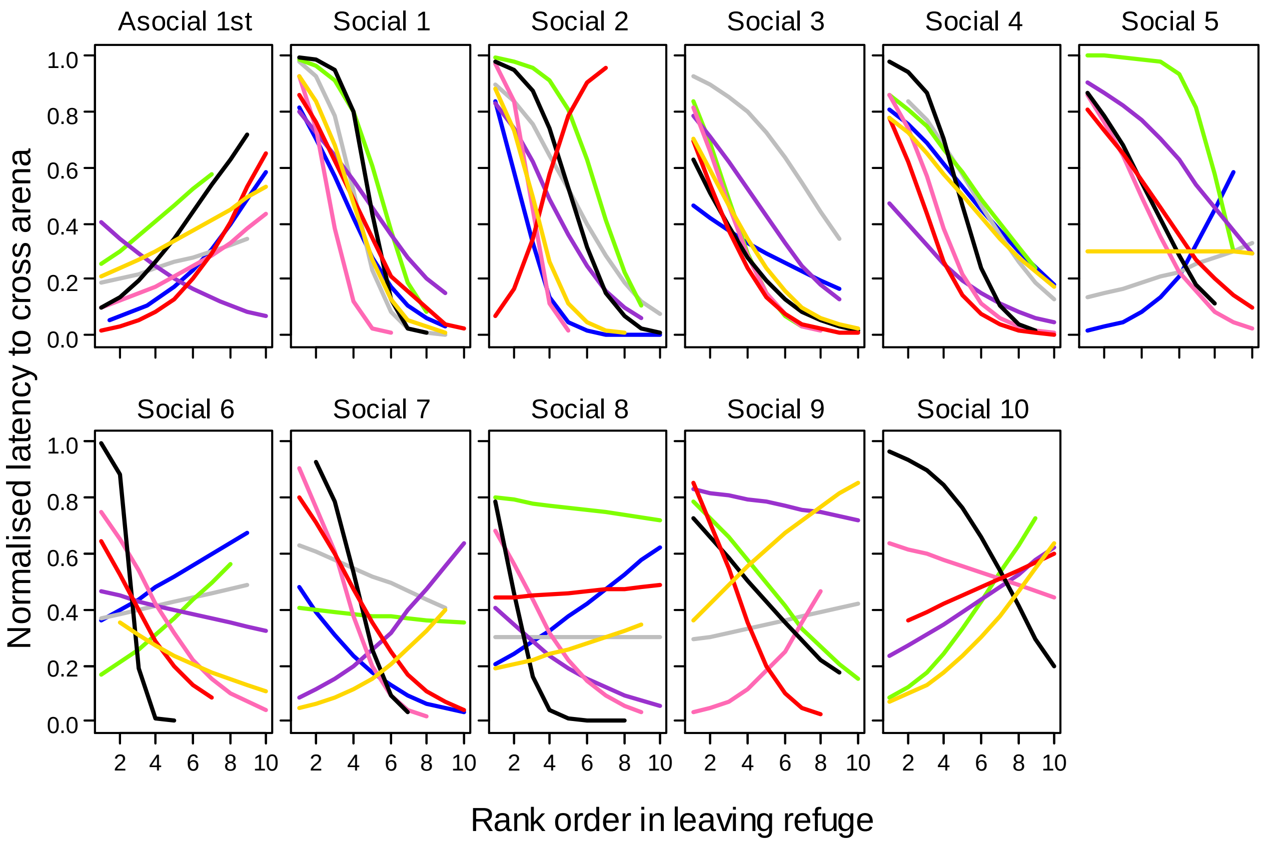

fig. S4. Collective decisions to cross the arena and its change over repeated trials on the first day of group trials. Shown is the relationship between the rank order of the fish in their latency to first leave the refuge and their latency to then cross the arena (normalised within that trial) for each trial order. Logistic regressions are fitted for each trial separately. Line colours depict each of the 8 groups of fish, as in Figure 4. Also shown are the model fits for the same analysis for each group applied to the latencies when fish were tested alone on the first day, before the group testing (Asocial 1st), which demonstrates the expected relationship if there were no social interactions between fish.

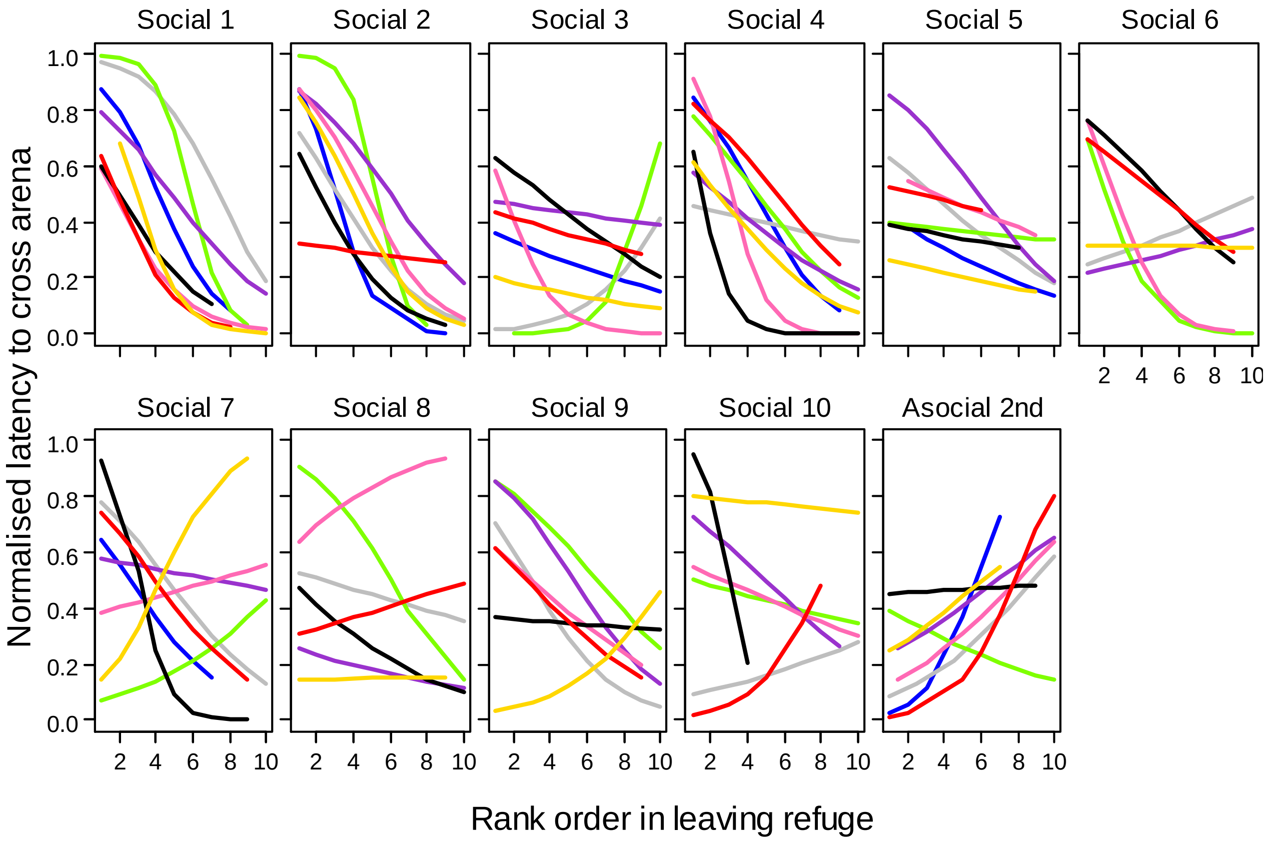

fig. S5. Collective decisions to cross the arena and its change over repeated trials on the second day of group trials. Plotting is as in Figure S4. Also shown are the model fits for the same analysis for each group applied to the latencies when fish were tested alone on the final day, after the group testing (Asocial 2nd).

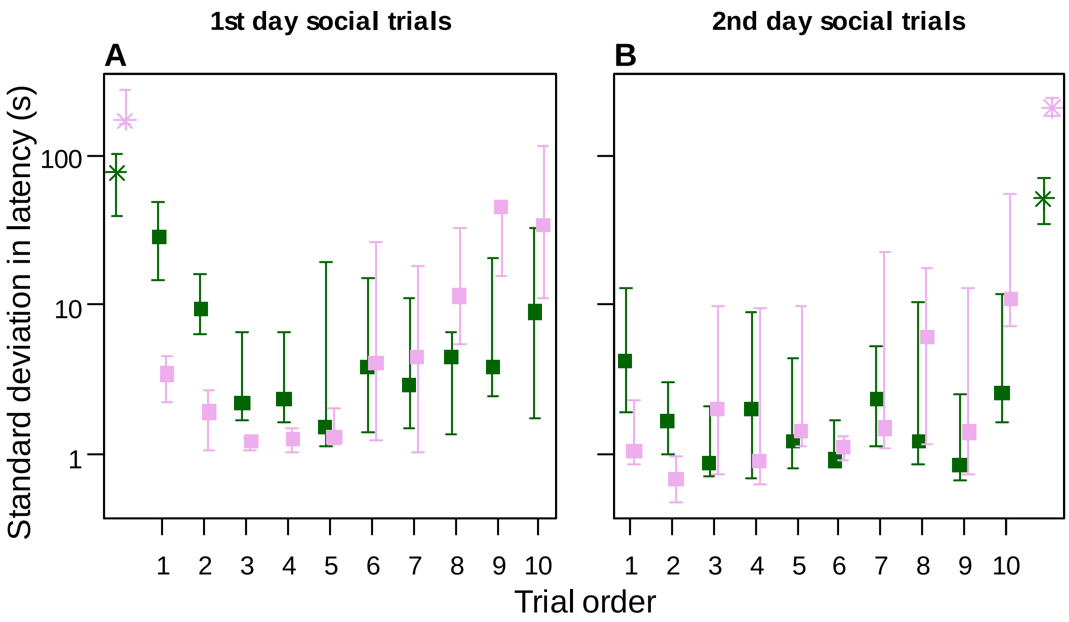

fig. S6. SD of latencies to leave the refuge (dark green) and the total time taken to reach the food (pink) within each trial as the group trials progressed each day. (**A**) The effect of trial order within the first day of group trials and (**B**) second day of group trials. Shown are medians (squares) and interquartile ranges. On both days, there was a significant interaction between the type of latency and the order of the trial (negative binomial GLMMs: trial order × leaving refuge or reaching the food, first day: $deviance_{6,7} = 16.01$, $P = 6.30\times10^{-5}$; second day: $deviance_{6,7} = 8.62$, $P = 0.0033$). Also shown are the standard deviations between fish in each group in leaving the refuge (dark green) and reaching the food (pink) when fish were tested alone before (in **A**) and after (in **B**) group trials (their medians are indicated by asterisks rather than squares). The standard deviations are plotted on a log10 scale.

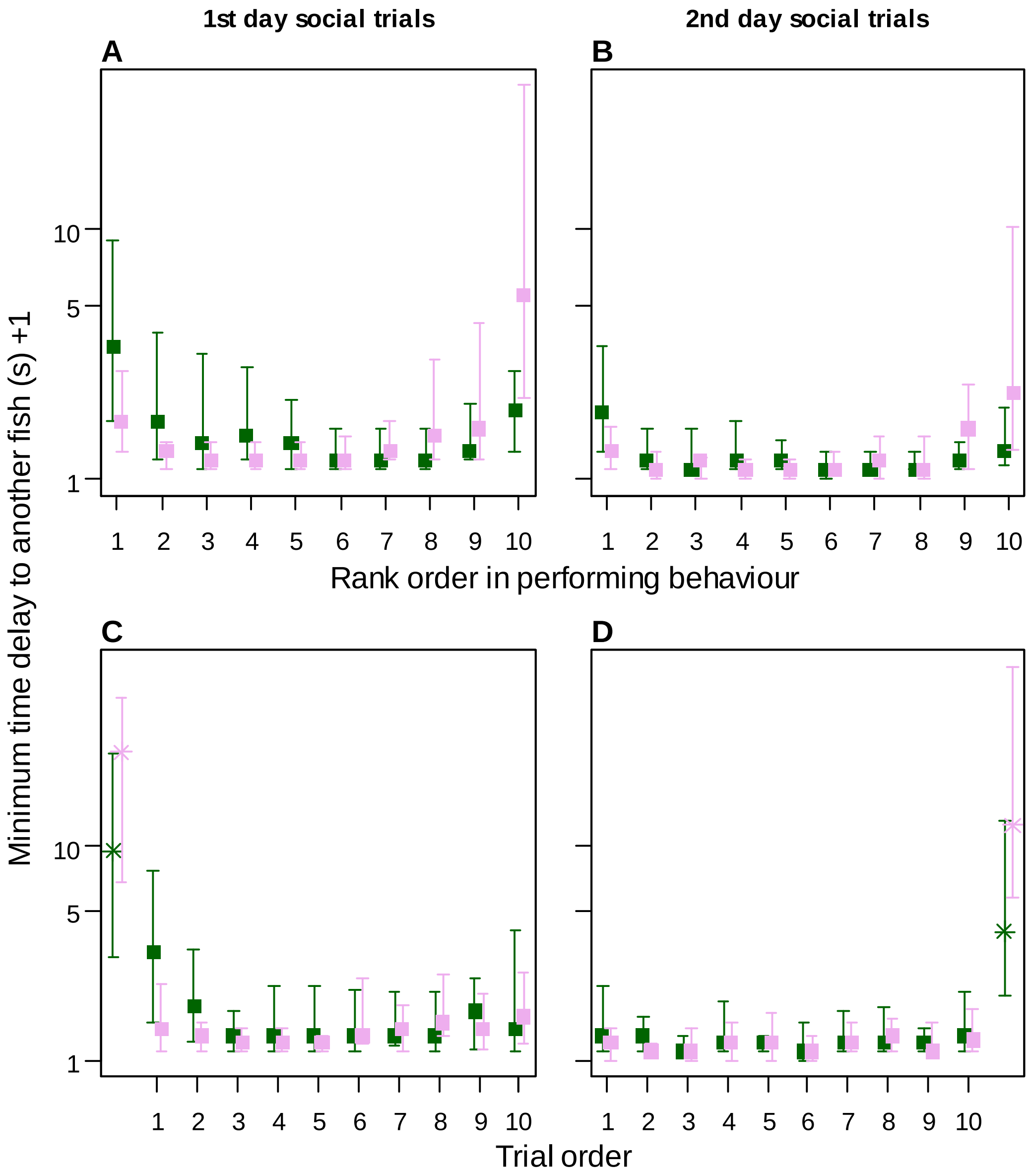

fig. S7. The minimum time delay from each fish to another fish in the trial to first leave the refuge (dark green) and reach the food (pink). (**A**) The effect of the rank order in performing each behaviour (whether leaving the refuge or reaching the food) on the first day of social trials, and the second (**B**). There is a significant interaction between rank order and type of latency on both days (negative binomial GLMM: rank order in trial × leaving refuge or reaching the food, first day: $\text{deviance}_{10,11} = 26.58$, $P = 2.53\times10^{-7}$; second day: $\text{deviance}_{10,11} = 19.32$, $P = 1.11\times10^{-5}$). (**C**) The effect of trial order on the first day of social trials, and the second (**D**). There is only a significant interaction on the first day of group trials (negative binomial GLMM: trial order × leaving refuge or reaching the food, first day: $\text{deviance}_{10,11} = 18.48$, $P = 1.72\times10^{-5}$; second day: $\text{deviance}_{10,11} = 1.98$, $P = 0.16$). Shown are medians and interquartile ranges. Also shown are the time delays between fish in each group in leaving the refuge (dark green) and reaching the food (pink) when fish were tested alone before (in **C**) and after (in **D**) group trials (their medians are indicated by asterisks rather than squares). The time delays are plotted on a log10 scale.

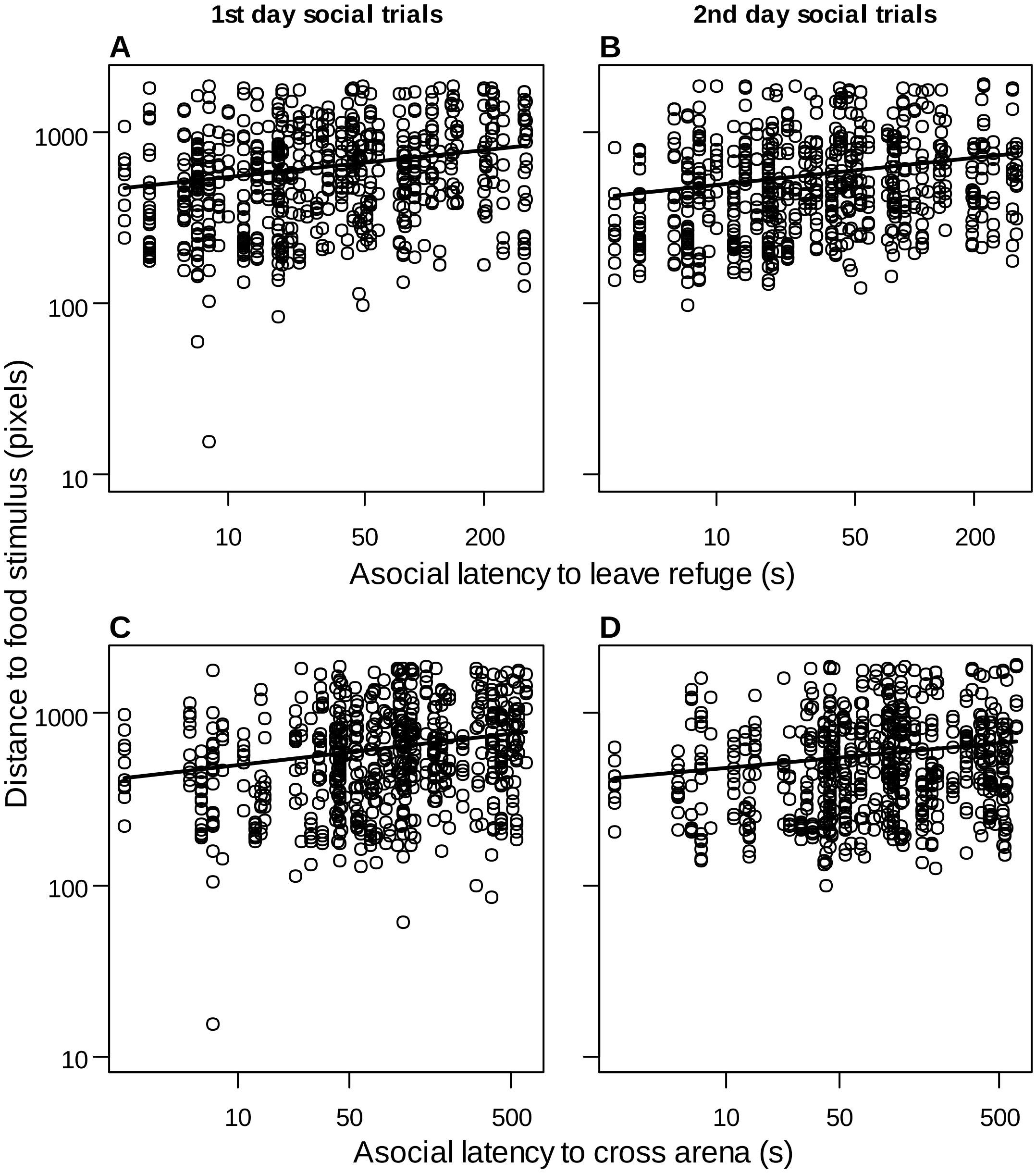

fig. S8. Relationship between latencies of fish tested alone and their distances from the food stimulus in each group trial when the first fish reaches the food. (**A,B**) The relationship on the first (**A**) and second (**B**) days of group trials for the latency to leave the refuge, and (**C,D**) for the latency to cross the arena on first (**C**) and second (**D**) days of group trials. Black lines show the fitted relationship from the GLMMs. The effect of boldness is statistically significant in all cases: latency to leave refuge when tested alone as measure of boldness: first day of group trials: $\text{deviance}_{9,10} = 15.72$, $P = 7.34\times10^{-5}$; second day: $\text{deviance}_{9,10} = 13.7$, $P = 0.000215$; latency to cross arena when tested alone: first day: $\text{deviance}_{9,10} = 15.72$, $P = 7.344\times10^{-5}$; second day: $\text{deviance}_{9,10} = 7.24$, $P = 0.00713$. Both asocial latencies and distances are plotted on a log10 scale.

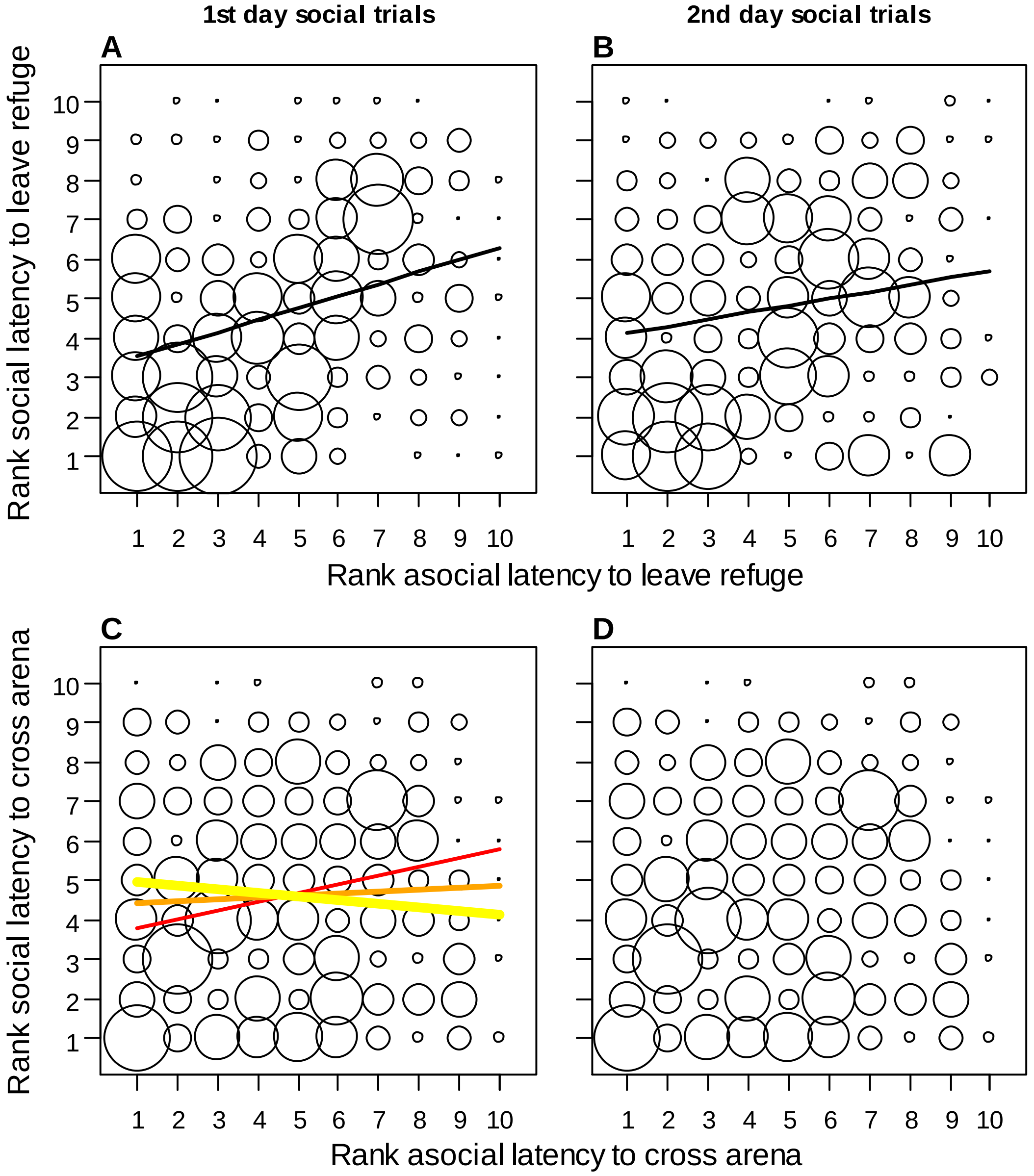

fig. S9. Relationship between the rank order in each group of latencies of fish tested alone (asocial) and in groups (social) for all group trials. Rank social latencies are plotted against corresponding rank asocial latencies for leaving the refuge (**A, B**) and crossing the arena (**C, D**), on the first (**A, C**) or second day (**B, D**) of the social trials. The circle diameter is proportional to the number of instances, with the largest being 19 cases (at (3,1) in panel **A**). The trendlines in **A, B** are fitted from the GLMMs showing significant main effects of the rank asocial latency to leave the refuge (LMM: first day: $\text{deviance}_{8,9} = 23.48$, $P = 1.262\times10^{-6}$, second day: $\text{deviance}_{8,9} = 7.94$, $P = 0.0048$). In **C**, they demonstrate the interaction between the rank asocial latency to leave the refuge and trial order ($\text{deviance}_{9,10} = 6.52$, $P = 0.010$), with the line thickness and colour representing the trial number (with the first being the thick yellow line, fifth the thinner orange line and the tenth the thin red line). Only these trendlines are shown for clarity (note the trendlines for the other orders are intermediate between these as trial order is a continuous variable). There was not any significant association between the rank asocial and social latencies in **D**, thus trendlines are omitted.

table S1. Summaries of the statistical models. Shown are coefficient estimates, their standard errors, their Z score, and the corresponding P value. (A) Shows the results for analyses of the latencies when tested alone and the first trial of each group testing day. In (B), (F) and (G) the sample sizes are the same within each day as cases where fish did not cross the arena were also removed from the corresponding analysis of their latency to leave the refuge for that day. In all models the group is a random effect, and fish ID is also included when there are repeated measures for each fish (all models except C and D). All statistically significant (at P<0.05) terms are in bold.

**Test and response variable**	**Explanatory variables**	**Estimate**	**S.E.**	**z value**	**P value**
**A: Effect of behaviour (leaving refuge/crossing arena), sociality (alone/group) and day (first/second) on latencies**					
Neg. bin. GLMM	Behaviour x Sociality x Day	-2636	0.3722	-0.71	0.47882
	Sociality x Day	-0.9947	0.2765	-3.6	**0.00032**
Latencies	Behaviour x Day	-0.6231	0.2668	-2.34	**0.01951**
n = 602	Behaviour x Sociality	1.4388	0.2591	5.55	**2.8×10^{-8}**
	Standard body length	-0.0442	0.0377	-1.17	0.24194
	Side of the pen	0.3043	0.337	0.9	0.3666
	Day	0.5022	0.1933	2.6	**0.00939**
	Sociality	-1.2938	0.1956	-6.61	**3.8×10^{-11}**
	Behaviour	6.8183	1.6777	4.06	**0.00087**

B: Effect of asocial latencies (i.e. boldness), trial order and whether a fish fed on latencies in group trials

Neg. bin. GLMM<br><br>Social latency to leave refuge (day 2)<br>n = 609	Trial order x log10 Asocial latency to leave refuge	-1.2052	0.4736	-2.54	**0.011**
	Linear effect of trial order	1.1381	1.7677	0.64	0.52
	Quadratic effect of trial order	9.9194	0.6147	14.96	**$<2\times10^{-16}$**
	log10 Asocial latency to leave refuge	0.0546	0.023	2.37	**0.018**
	Whether fed in previous trial	-0.2753	0.0561	-4.91	**9.1×10^{-7}**
	Standard body length	0.0178	0.0194	0.91	0.361
	Side of the pen	0.8079	0.5088	1.59	0.112
Neg. bin. GLMM<br><br>Social latency to leave refuge (day 3)<br>n = 625	Trial order x log10 Asocial latency to leave refuge	-0.10202	0.48053	-0.21	0.8319
	Linear effect of trial order	2.92734	1.7781	1.65	0.0997
	Quadratic effect of trial order	5.2582	0.61407	8.56	**$<2\times10^{-16}$**
	log10 Asocial latency to leave refuge	0.02462	0.02256	1.09	0.2752
	Whether fed in previous trial	-0.16869	0.05646	-2.99	**0.0028**
	Standard body length	0.00185	0.0181	0.1	0.9185
	Side of the pen	1.18092	0.6768	1.74	0.081
Neg. bin. GLMM<br><br>Social latency to cross arena (day 2)<br>n = 609	Trial order x log10 Asocial latency to cross arena	-0.1145	0.8131	-0.14	0.88797
	Linear effect of trial order	12.7379	3.7248	3.42	**0.00063**
	Quadratic effect of trial order	12.2315	1.1647	10.5	**$<2\times10^{-16}$**
	log10 Asocial latency to cross arena	0.0706	0.0422	1.67	0.094
	Whether fed in previous trial	-0.3216	0.0986	-3.26	**0.00111**
	Standard body length	-0.0668	0.0396	-1.69	0.09122
	Side of the pen	0.4656	0.493	0.94	0.34492
Neg. bin. GLMM<br><br>Social latency to cross arena (day 3)<br>n = 625	Trial order x log10 Asocial latency to cross arena	0.77271	0.81122	0.95	0.341
	Linear effect of trial order	8.11726	3.7186	2.18	**0.029**
	Quadratic effect of trial order	11.38264	1.0996	10.35	**$<2\times10^{-16}$**
	log10 Asocial latency to cross arena	0.00921	0.03716	0.25	0.804
	Whether fed in previous trial	-0.2329	0.1006	-2.32	**0.021**
	Standard body length	-0.00398	0.0319	-0.12	0.901
	Side of the pen	1.56607	0.94022	1.67	0.096
Neg. bin. GLMM<br><br>Social total time taken (day 2)<br>n = 609	Trial order x log10 Asocial total time taken	-0.00475	0.00761	-0.62	0.53
	Linear effect of trial order	3.79266	2.6326	1.44	0.15
	Quadratic effect of trial order	6.5795	0.63977	10.28	**$<2\times10^{-16}$**
	log10 Asocial latency to leave refuge and cross arena	0.05608	0.04672	1.2	0.23
	Whether fed in previous trial	-0.26912	0.05673	-4.74	**2.1×10^{-6}**
	Standard body length	-0.00212	0.0199	-0.11	0.92
	Side of the pen	0.34067	0.36386	0.94	0.34
Neg. bin. GLMM<br><br>Social total time taken (day 3)<br>n = 625	Trial order x log10 Asocial total time taken	-0.00564	0.00816	-0.69	0.4895
	Linear effect of trial order	8.77798	2.9867	2.94	**0.0033**
	Quadratic effect of trial order	3.34108	0.77554	4.31	**1.6×10^{-5}**
	Log10 Asocial latency to leave refuge and cross arena	0.04986	0.04952	1.01	0.3139
	Whether fed in previous trial	-0.15602	0.06496	-2.4	**0.0163**

Standard body length	-0.01201	0.01972	-0.61	0.5425
Side of the pen	0.99848	0.49321	2.02	**0.0429**

**C: Effects on the deviance of the logistic regression fit between normalised time taken and rank in group**					
Poisson GLMM<br><br>Link-logit deviance (day 2) n = 77	Trial order	0.1424	0.027	5.28	**1.3×10^{-7}**
	Side of the pen	-0.0871	0.2727	-0.32	0.75
Poisson GLMM<br><br>Link-logit deviance (day 3) n = 76	Trial order	0.0658	0.222	2.96	**0.003**
	Side of the pen	-0.0976	0.127	-0.77	0.442

**D: Effect of behaviour (leaving refuge/crossing arena) and trial order on the S.D. of latencies in a group**					
Neg. bin. GLMM<br><br>Standard deviation of latency (day 2) n = 154	Behaviour x Trial order	0.2381	0.0584	4.08	**4.6×10^{-5}**
	Behaviour	-1.3142	0.3551	-3.7	**0.00022**
	Trial order	-0.033	0.0416	-0.79	0.42679
	Side of the pen	0.243	0.2458	0.99	0.3227
Neg. bin. GLMM<br><br>Standard deviation of latency (day 3) n = 152	Behaviour x Trial order	0.1549	0.0521	2.98	**0.00292**
	Behaviour	-0.6242	0.3214	-1.94	0.05211
	Trial order	0.0303	0.0386	0.78	0.43317
	Side of the pen	0.8017	0.4812	1.67	0.09573

**E: Effect of leaving refuge or crossing arena, trial order and rank on minimum time delay to another fish**					
Neg. bin. GLMM<br><br>Time delay between fish (day 2) n = 1433	Trial order x Behaviour	0.0978	0.0227	4.3	**1.7×10^{-5}**
	Behaviour x Rank	0.1275	0.0247	5.16	**2.5×10^{-7}**
	Trial order	-0.042	0.0156	-2.7	**0.00691**
	Behaviour	-1.3529	0.1905	-7.1	**1.2×10^{-12}**
	Rank	-0.0515	0.0151	-3.41	**0.00065**
	Standard body length	-0.0192	0.0177	-1.08	0.27948
	Side of the pen	-0.0894	0.1295	-0.69	0.49035
Neg. bin. GLMM<br><br>Time delay between fish (day 3) n = 1446	Trial order x Behaviour	0.03257	0.02309	1.41	0.1583
	Behaviour x Rank	0.11094	0.02521	4.4	**1.1×10^{-5}**
	Trial order	0.00682	0.01614	0.42	0.6727
	Behaviour	-0.83171	0.019723	-4.22	**2.5×10^{-5}**
	Rank	-0.02766	0.01611	-1.72	0.086
	Standard body length	-0.05523	0.02209	-2.5	**0.0124**
	Side of the pen	-0.36206	0.1215	-2.98	**0.0029**

F: Relationship between distance to stimulus in group trials and latencies in asocial trials (i.e. boldness)					
Neg. bin. GLMM<br><br>Distance to the stimulus (day 2)<br>n = 658	Trial order x log10 Asocial latency to leave refuge	0.3792	0.436	0.87	0.38441
	log10 Asocial latency to leave refuge	0.1207	0.03	4.03	**5.7×10^{-5}**
	Linear effect of trial order	-5.7668	1.5844	-3.64	**0.00027**
	Quadratic effect of trial order	0.5094	0.5531	0.92	0.35706
	Whether fed in previous trial	-0.1941	0.0498	-3.89	**9.8×10^{-5}**
	Side of the pen	-0.0886	0.0741	-1.2	0.23187
	Standard body length	0.0339	0.0125	2.71	**0.00664**
Neg. bin. GLMM<br><br>Distance to the stimulus (day 3)<br>n = 652	Trial order x log10 Asocial latency to leave refuge	-0.17203	0.44159	-0.39	0.69686
	log10 Asocial latency to leave refuge	0.12441	0.03244	3.84	**0.00013**
	Linear effect of trial order	-1.67423	1.6275	-1.03	0.30362
	Quadratic effect of trial order	0.23176	0.55306	0.42	0.67518
	Whether fed in previous trial	-0.06867	0.05279	-1.3	0.19332
	Side of the pen	-0.00979	0.12429	-0.08	0.93724
	Standard body length	0.00135	0.01893	0.07	0.94326
Neg. bin. GLMM<br><br>Distance to the stimulus (day 2)<br>n = 658	Trial order x log10 Asocial latency to cross arena	-0.55	0.3728	-1.48	0.1401
	log10 Asocial latency to cross arena	0.0948	0.0235	4.03	**5.6×10^{-5}**
	Linear effect of trial order	-1.3363	1.7367	-0.77	0.4416
	Quadratic effect of trial order	0.6984	0.4852	1.44	0.15
	Whether fed in previous trial	-0.1951	0.0434	-4.5	**6.9×10^{-6}**
	Side of the pen	-0.072	0.0612	-1.18	0.2392
	Standard body length	0.0332	0.0106	3.14	**0.0017**
Neg. bin. GLMM<br><br>Distance to the stimulus (day 3)<br>n = 652	Trial order x log10 Asocial latency to cross arena	-0.69475	0.40332	-1.72	0.085
	log10 Asocial latency to cross arena	0.06626	0.02801	2.37	**0.018**
	Linear effect of trial order	1.13949	1.8641	0.61	0.541
	Quadratic effect of trial order	-0.01798	0.52895	-0.03	0.973
	Whether fed in previous trial	-0.0744	0.04918	-1.51	0.13
	Side of the pen	-0.0378	0.10031	-0.37	0.708
	Standard body length	0.00337	0.0162	0.21	0.835

G: Effect of rank asocial latency, trial order and whether a fish fed on rank latency in group trials					
LMM<br><br>Rank social latency to leave refuge (day 2)<br>n = 609	Trial order x Rank asocial latency to leave refuge	0.0077	0.01229	0.63	0.5309
	Whether fed in previous trial x Trial order	-0.00169	0.0639	-0.03	0.9789
	Rank asocial latency to leave refuge	0.30617	0.06152	4.98	**6.50×10^{-7}**
	Trial order	0.00798	0.03102	0.26	0.797
	Whether fed in previous trial	-0.32232	0.19363	-1.66	0.096
	Side of the pen	-0.1176	0.32224	-0.36	0.715
	Standard body length	0.09308	0.05631	1.65	0.098
LMM<br><br>Rank social latency to leave refuge (day 3)<br>n = 625	Trial order x Rank asocial latency to leave refuge	-0.00689	0.01239	-0.56	0.578
	Whether fed in previous trial x Trial order	-0.03687	0.06675	-0.55	0.581
	Rank asocial latency to leave refuge	0.175	0.0617	2.84	**0.0045**
	Trial order	-0.0375	0.0321	-1.17	0.2432
	Whether fed in previous trial	-0.1244	0.2115	-0.59	0.5564
	Side of the pen	-0.2115	0.3181	-0.66	0.5062
	Standard body length	0.1116	0.0556	2.01	**0.0445**
LMM<br><br>Rank social latency to cross arena (day 2)<br>n = 609	Trial order x Rank asocial latency to leave refuge	0.0346	0.0135	2.56	**0.01**
	Whether fed in previous trial x Trial order	-0.10498	0.07031	-1.49	0.135
	Rank asocial latency to leave refuge	-0.1266	0.0898	-1.41	0.159
	Trial order	-0.162	0.0713	-2.27	**0.023**
	Whether fed in previous trial	-0.2042	0.2093	-0.98	0.329
	Side of the pen	-0.0541	0.2804	-0.19	0.847
	Standard body length	0.1173	0.049	2.39	**0.017**
LMM<br><br>Rank social latency to cross arena (day 3)<br>n = 625	Trial order x Rank asocial latency to leave refuge	0.00933	0.01293	0.72	0.47
	Whether fed in previous trial x Trial order	-0.07593	0.06969	-1.09	0.28
	Rank asocial latency to leave refuge	0.0277	0.0572	0.48	0.628
	Trial order	-0.0414	0.034	-1.22	0.224
	Whether fed in previous trial	-0.2663	0.2187	-1.22	0.223
	Side of the pen	-0.295	0.2917	-1.01	0.312
	Standard body length	0.0846	0.0509	1.66	0.096

H: Effect of asocial latency (i.e. boldness) and trial order on whether a fish fed in each group trial					
Binomial GLMM<br><br>Whether fed in trial (day 2)<br>n = 711	Trial order x log10 Asocial latency to leave refuge	-0.0076	0.0265	-0.29	0.77
	log10 Asocial latency to leave refuge	-0.3273	0.1269	-2.58	**0.0099**
	Linear effect of trial order	2.6014	2.3603	1.1	0.2704
	Quadratic effect of trial order	-0.5541	2.3586	-0.23	0.8143
	Side of the pen	-0.1786	0.3085	-0.58	0.5625
	Standard body length	-0.0304	0.0535	-0.57	0.5694
Binomial GLMM<br><br>Whether fed in trial (day 3)<br>n = 700	Trial order x log10 Asocial latency to leave refuge	0.000724	0.029474	0.02	0.98
	log10 Asocial latency to leave refuge	-0.5098	0.1512	-3.37	**0.00075**
	Linear effect of trial order	-1.6284	2.6143	-0.62	0.53336
	Quadratic effect of trial order	0.7985	2.5742	0.31	0.75642
	Side of the pen	-0.3574	0.3603	-0.99	0.32122
	Standard body length	-0.0111	0.0625	-0.18	0.85925
Binomial GLMM<br><br>Whether fed in trial (day 2)<br>n = 711	Trial order x log10 Asocial latency to cross arena	-0.00081	0.024974	-0.03	0.97
	log10 Asocial latency to cross arena	-0.2933	0.12	-2.44	**0.015**
	Linear effect of trial order	2.5545	2.3594	1.08	0.279
	Quadratic effect of trial order	-0.6077	2.3582	-0.26	0.797
	Side of the pen	-0.2033	0.3101	-0.66	0.512
	Standard body length	-0.0327	0.0538	-0.61	0.543
Binomial GLMM<br><br>Whether fed in trial (day 3)<br>n = 700	Trial order x log10 Asocial latency to cross arena	0.0446	0.0286	1.56	0.1192
	log10 Asocial latency to cross arena	-0.4034	0.146	-2.76	**0.0057**
	Linear effect of trial order	-1.6637	2.6149	-0.64	0.5246
	Quadratic effect of trial order	0.7913	2.5748	0.31	0.7586
	Side of the pen	-0.4024	0.3699	-1.09	0.2766
	Standard body length	-0.0135	0.0641	-0.21	0.8326

table S2. Frequencies of fish trial leaving the refuge, crossing the arena, and consuming bloodworm before the end of each trial. There are 10 fish in each group trial.

Day	Group	Order	N leaving the refuge	N crossing arena	N consuming food
2	1	1	10	10	7
2	1	2	10	8	7
2	1	3	10	7	6
2	1	4	10	9	7
2	1	5	10	8	7
2	1	6	10	6	5
2	1	7	10	10	6
2	1	8	10	10	8
2	2	1	10	10	8
2	2	2	10	10	8
2	2	3	10	9	6
2	2	4	10	9	5
2	2	5	10	10	6
2	2	6	10	6	4
2	2	7	10	8	5
2	2	8	10	10	6
2	2	9	10	10	5
2	3	1	10	9	7
2	3	2	9	9	8
2	3	3	9	7	5
2	3	4	10	8	6
2	3	5	9	8	6
2	3	6	10	8	5
2	3	7	10	8	8
2	3	8	10	7	6
2	3	9	10	9	7
2	3	10	10	9	8
2	4	1	10	9	7
2	4	2	10	9	7
2	4	3	10	10	6
2	4	4	10	10	8
2	4	5	10	10	7
2	4	6	10	10	7
2	4	7	10	10	8
2	4	8	10	9	8
2	4	9	10	9	8
2	4	10	10	9	5
2	5	1	10	9	4

2	5	2	10	8	6
2	5	3	10	9	6
2	5	4	10	10	6
2	5	5	10	9	7
2	5	6	10	10	5
2	5	7	10	8	5
2	5	8	10	9	7
2	5	9	10	7	5
2	5	10	10	9	8
2	6	1	10	9	6
2	6	2	10	9	7
2	6	3	10	10	7
2	6	4	10	9	5
2	6	5	10	8	7
2	6	6	10	8	7
2	6	7	10	6	5
2	6	8	10	8	7
2	6	9	10	8	8
2	6	10	10	10	4
2	7	1	10	5	3
2	7	2	10	8	4
2	7	3	10	10	9
2	7	4	10	10	7
2	7	5	10	9	7
2	7	6	10	7	7
2	7	7	10	10	7
2	7	8	10	8	8
2	7	9	10	7	6
2	7	10	10	8	7
2	8	1	10	9	5
2	8	2	9	8	7
2	8	3	10	10	7
2	8	4	10	8	6
2	8	5	10	9	8
2	8	6	10	7	6
2	8	7	10	8	6
2	8	8	10	8	6
2	8	9	10	8	7
2	8	10	10	10	8
3	1	1	10	10	6
3	1	2	10	7	5
3	1	3	10	9	8
3	1	4	10	8	6

3	1	5	10	9	7
3	1	7	10	8	6
3	2	1	10	10	5
3	2	2	10	10	7
3	2	3	10	10	6
3	2	4	10	10	8
3	2	5	10	10	9
3	2	6	10	10	8
3	2	7	10	9	5
3	2	8	10	10	7
3	2	9	10	10	10
3	2	10	10	10	4
3	3	1	10	9	8
3	3	2	10	8	5
3	3	3	10	8	5
3	3	4	10	10	8
3	3	5	10	10	7
3	3	6	10	10	7
3	3	7	10	9	5
3	3	8	10	9	7
3	3	9	10	8	7
3	3	10	10	9	10
3	4	1	10	10	8
3	4	2	10	10	8
3	4	3	10	10	6
3	4	4	10	9	8
3	4	5	10	9	6
3	4	6	10	10	6
3	4	7	10	9	8
3	4	8	10	8	7
3	4	9	10	10	7
3	4	10	10	8	8
3	5	1	10	10	9
3	5	2	10	10	9
3	5	3	10	10	9
3	5	4	10	10	7
3	5	5	10	7	5
3	5	6	10	8	7
3	5	7	10	9	7
3	5	8	10	7	5
3	5	9	10	6	6
3	5	10	10	9	7
3	6	1	10	10	8

3	6	2	10	9	7
3	6	3	10	10	8
3	6	4	10	9	7
3	6	5	10	9	7
3	6	6	10	8	7
3	6	7	10	9	8
3	6	8	10	9	9
3	6	9	10	9	7
3	6	10	10	10	7
3	7	1	10	9	7
3	7	2	10	9	7
3	7	3	9	9	7
3	7	4	9	9	9
3	7	5	10	9	7
3	7	6	9	9	6
3	7	7	9	9	6
3	7	8	10	7	6
3	7	9	9	8	7
3	7	10	10	8	7
3	8	1	10	9	8
3	8	2	10	10	7
3	8	3	10	10	9
3	8	4	10	10	8
3	8	5	10	9	8
3	8	6	10	9	7
3	8	7	10	8	7
3	8	8	10	9	6
3	8	9	10	10	8
3	8	10	10	10	9
